# Long non-coding RNAs in B-cell malignancies: a comprehensive overview

**DOI:** 10.18632/oncotarget.17303

**Published:** 2017-04-20

**Authors:** Lucia Nobili, Domenica Ronchetti, Elisa Taiana, Antonino Neri

**Affiliations:** ^1^ Department of Oncology and Hemato-Oncology, Università degli Studi di Milano, Hematology, Fondazione Cà Granda IRCCS Policlinico, Milano, Italy

**Keywords:** long non-coding RNAs, B-cell differentiation, B-cell malignancies, expression profiling, transcription regulation

## Abstract

B-cell malignancies constitute a large part of hematological neoplasias. They represent a heterogeneous group of diseases, including Hodgkin's lymphoma, most non-Hodgkin's lymphomas (NHL), some leukemias and myelomas. B-cell malignancies reflect defined stages of normal B-cell differentiation and this represents the major basis for their classification. Long non-coding RNAs (lncRNAs) are non-protein-coding transcripts longer than 200 nucleotides, for which many recent studies have demonstrated a function in regulating gene expression, cell biology and carcinogenesis. Deregulated expression levels of lncRNAs have been observed in various types of cancers including hematological malignancies. The involvement of lncRNAs in cancer initiation and progression and their attractive features both as biomarker and for therapeutic research are becoming increasingly evident. In this review, we summarize the recent literature to highlight the status of the knowledge of lncRNAs role in normal B-cell development and in the pathogenesis of B-cell tumors.

## INTRODUCTION

**Figure 1:**
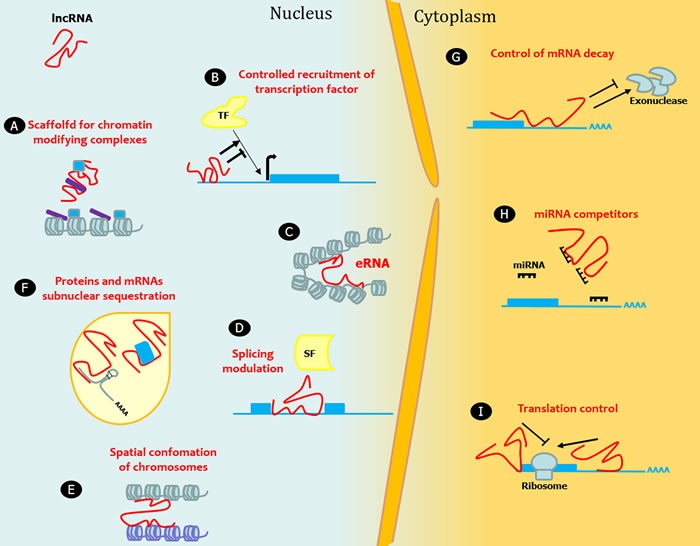
LncRNAs in epigenetic, transcriptional and translational regulation Nine mechanisms of epigenetic, transcriptional and translational regulation by lncRNAs are shown. **A.** LncRNAs act as a transcriptional guide by recruiting chromatin-modifying enzymes to target genes with activator (blue) or repressive (violet) histone marks. **B.** LncRNAs control the recruitment of transcription factors (TF) and core components of the transcriptional machinery. **C.** LncRNAs act as a transcriptional guide by accosting enhancer elements. **D.** LncRNAs bind mRNAs and splicing factors (SF) and modulate splicing processing. **E.** LncRNAs participate in the higher order organization of the nucleus by mediating spatial conformation of chromosomes and **F.** as structural components for the formation and function of nuclear bodies. **G.** LncRNAs modulate mRNA decay protecting mRNA from degradation or, alternatively, mediating the recruitment of degradation machinery. **H.** LncRNAs can act as miRNA sponges, thus favoring the expression of the mRNAs targeted by the sequestered miRNA. **I.** LncRNAs control translation rates favoring or inhibiting polysome loading to mRNAs.

LncRNAs are a highly heterogeneous group of RNA molecules longer than 200 nucleotides accounting for more than half of the mammalian non-coding transcriptome. The definitive number of human lncRNA transcripts is still unknown; the repository LNCipedia currently contains 118,777 human annotated lncRNAs, with many loci generating multiple transcripts [1]. There is an exponential interest in investigating lncRNAs as considerable evidence suggests a huge impact of lncRNAs on several molecular mechanisms. LncRNA expression is regulated during development, and can be tissue- and cell-type specific. The mechanisms underlying the function of most lncRNAs are not fully understood. The structural versatility of lncRNAs might be crucial to form binding sites for the interaction with proteins, DNA, and other RNA molecules by serving as guides, tethers, decoys, and scaffolds. From a functional point of view, lncRNAs regulate gene expression at multiple levels including transcriptional, post-transcriptional, and chromatin modification (reviewed in [2–8]). For instance, they can directly act, either in *cis* or in *trans*, on the genomic DNA to regulate gene expression. To this end, they may recruit chromatin-modifying complexes dictating the conformation of heterochromatin that represses transcription, or they may activate transcription by either triggering enhancer regions or inducing three dimensional chromatin conformation changes. Other lncRNAs can interact with transcription factors and RNA-binding proteins to indirectly regulate transcription. With a different mechanism, lncRNAs can indirectly alter gene expression by competing with mRNAs for miRNA binding. LncRNAs can also act regulating mRNA processing events such as splicing, editing, localization, translation and turnover/degradation (Figure 1).

LncRNAs expression is involved in oncogenic or tumor suppressor processes [9]. Moreover, it has been reported in many types of tumors that dysregulation of distinct lncRNAs promotes cancerogenesis, disease progression, and metastasis [10, 11]. An increasing number of evidence suggests that lncRNAs are leading actors both in normal and malignant hematopoiesis (reviewed in [12–16]).

We have recently reviewed the current knowledge about lncRNAs involvement in normal hematopoiesis and in hematological tumors. The data available thus far suggest that several lncRNAs may have a prospective clinical significance in the diagnosis, prognosis, and therapy of these malignancies.

An additional chapter deserve B-cell malignancies that constitute a large part of hematological neoplasias. They represent a heterogeneous group of diseases, including Hodgkin's lymphoma, most non-Hodgkin's lymphomas (NHL), some leukemias and myelomas. B-cell malignancies mirror specific stages of normal B-cell differentiation and this represents the major basis for their classification. This review focuses on lncRNA involvement in normal human B-cell differentiation and in the pathogenesis and natural history of B-cell tumors.

## B-CELL DIFFERENTIATION

In adults, B-cell differentiation begins within bone marrow (BM) and concludes in the peripheral secondary lymphoid organs, such as lymph nodes, tonsils, and spleen.

B-cell differentiation in the BM passes through different cellular stages including the common lymphoid progenitor, pro-B cell, pre-B cell and immature-B cell stages [17]. These stages are demarcated by consecutive steps of the process of somatic recombination that assembles different gene portions within the heavy or light chain loci to produce functional B cell receptor (BCR)/immunoglobulin (Ig) genes. Specifically, the somatic rearrangement of the Variable (V), Diversity (D) and Joining (J) gene segments with the Constant (C) region exons gives rise to heavy chains Igs with unique antigenic specificity. The light chains rearrange in a very similar way, but do not have D gene segments. Fruitful assembly of heavy and light chains results in the expression of complete IgM molecule at the surface of immature B-cell that, once tested for auto-reactivity by the immune system, leaves the BM and migrates *via* the blood and the lymph to the secondary lymphoid organs.

In the periphery B-cells differentiate to the mature or naïve stage in the absence of any contact with exogenous antigens [18]. The encounter with an antigen establishes the second phase of B-cell maturation. More specifically, once bound to an antigen, the B-cell enters into primary lymphoid follicles of peripheral lymphoid tissues resulting in the formation of germinal centers (GCs). In the GC a dark and a light zone can be distinguished. The dark zone is formed by centroblasts (CBs), which are rapidly dividing B-cells that achieve further diversification of the Ig repertoire through the somatic hypermutation of the V regions of Ig genes [19]. CBs eventually differentiate into non-cycling centrocytes (CCs), which constitute the light zone of the GC. The CCs are able to generate new antibody variants that are selected according to their affinity to the cognate antigen, ensuring increased affinity between the Ig and the antigen. In addition, a fraction of CCs can undergo Ig class switching by somatic DNA recombination, thus generating antibodies with different effector functions. Finally, B-cells leave the GC rapidly differentiating into long-lived PCs or memory B-cells, otherwise they might re-enter the dark zone and reiterate the cycle [20–22].

### LncRNAs IN B-CELL DIFFERENTIATION

**Figure 2:**
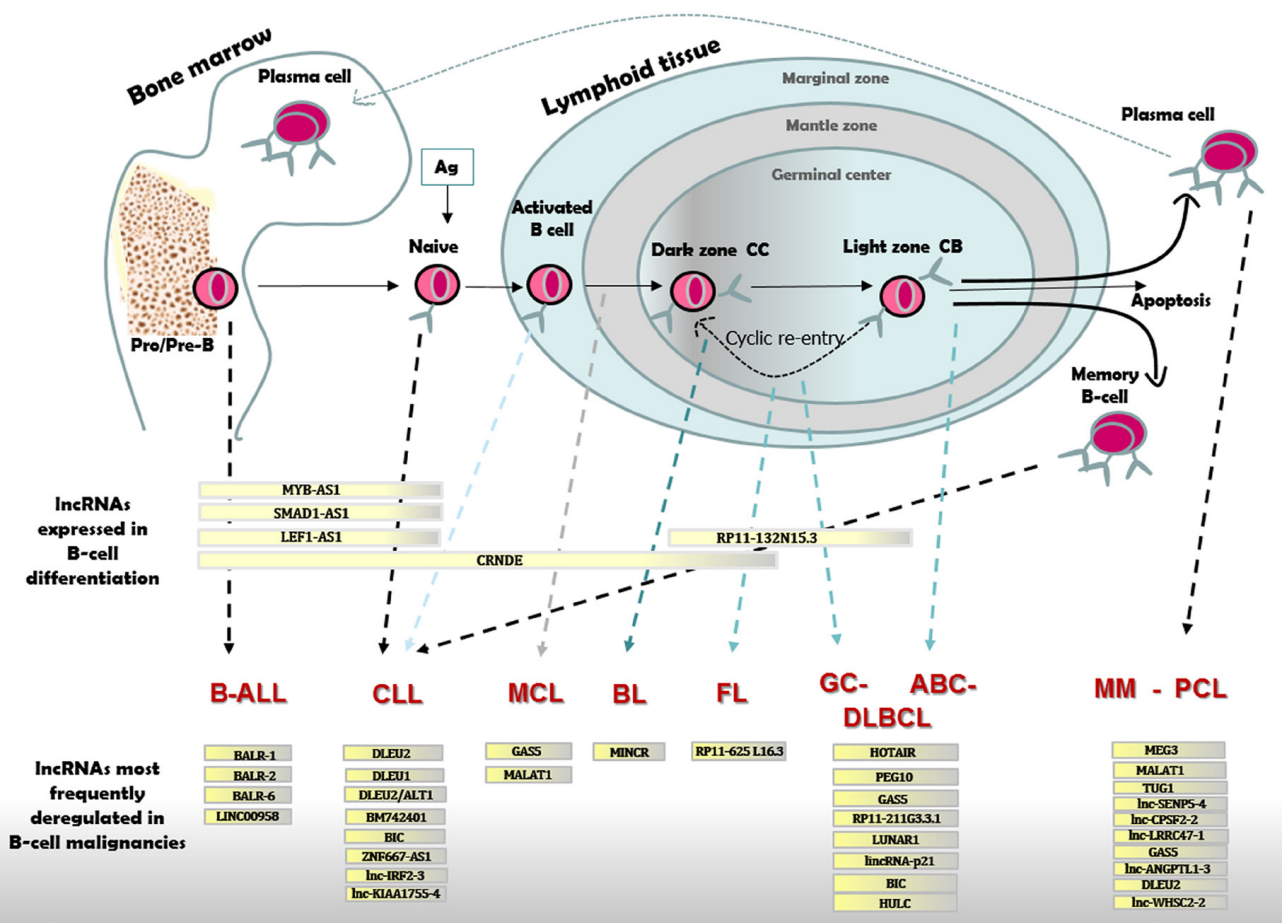
LncRNAs expressed in B-cell differentiation or pathology B-cell differentiation in BM and lymphoid tissue with distinct types of B-cell malignancies originating from cells blocked at different stages of maturation. B-ALL cells resemble Pro/Pre-B lymphocytes; CLL originate from naïve or activated unmutated IGHV B-cells in the marginal zone or from mutated IGHV committed memory B-cell; MCL are like B-cells from the mantle zone; BL resemble dark zone B-cells; FL and GC-DLBCL originate from light zone B-cells; ABC-DLBCL show characteristic of late GC B-cells; MM and PCL originate from mature PCs. Differentially expressed lncRNAs are reported for each stage of B-cell maturation, and for distinct B-cell malignancies as compared to normal B-cell.

The role of lncRNAs in normal human B-cell differentiation remains to be fully elucidated (see Figure 2 and Table 1 for details on lncRNAs discussed in the text below).

**Table 1:** LncRNAs with roles in normal B-cells

lncRNA(s)/alias	Stage of B-cell	Genome location	Function	Molecular mechanism	Reference(s)
**MYB-AS1, SMAD1-AS1, LEF1-AS1**	pre-B1 and pre-B2 cells	6q23.3, 4q31.21, 4q25	Involvement in early B-cell development	Antisense transcripts to TFs with well-known roles in early B-cells	[23]
**CRNDE**	pre-B1, pre-B2, CBs and, to a lesser extent, CCs	16q12.2	Involvement in mitotic cell cycle related processes	Promotion of the metabolic changes by which cancer cells switch to aerobic glycolysis	[23, 25–27]
**RP11-132N15.3/lnc-BCL6-3**	CBs and, to a lesser extent, CCs	3q27.3	Involvement in the modulation of the GC reaction	LincRNA possibly regulating the transcriptional repressor BCL-6	[23]

*Abbreviations*: CC, centrocyte; CB, centroblast; GC, germinal center

The identification of lncRNAs specifically expressed during different stages of human B-cell development has been recently analyzed by Petri et al. [23]. Eleven distinct B-cell subsets separated by flow cytometry, including pre-B1, pre-B2, immature, naïve, memory, and PCs from BM biopsies, and naïve, CB, CC, memory, and plasmablast cells from tonsil tissues, respectively, were subjected to microarray expression analysis. This study pointed out numerous lncRNAs belonging to well-defined gene networks linked to precise steps of cellular differentiation. Notably, among sequences expressed mainly in pre-B1 and pre-B2 cells, Petri et al. identified several lncRNAs, including antisense transcripts (*SMAD1-AS1*, *MYB-AS1*, and *LEF1-AS1*) to transcription factors known to have a function in early B-cells. Although the role of these lncRNAs in B-cell development is still unknown, their crucial positions in the gene co-expression network analysis suggest key functions in the early stages of cellular differentiation. In the co-expression module including genes that display highest expression levels in pre-B1, pre-B2 cells, and CBs, and low ones in CCs, were found overexpressed genes involved in processes related to mitosis and cell cycle control, consistently with the high proliferative activity of both pre-B cells and GC CBs [24]. Among lncRNAs showing strong and highly significant correlation to this module, the authors focused on *CRNDE* (colorectal neoplasia differentially expressed), encoded by a gene located on chromosome 16 and expressing multiple splice variants [25, 26]. Initially identified as highly expressed in colorectal cancer [25], *CRNDE* is also upregulated in many other types of solid tumors and in leukemias and highly expressed in the early stages of human development (see [26] for a review). A recent study has shown that in tumoral cells *CRNDE* expression favors the metabolic switch to aerobic glycolysis [27], which is required during rapid cell proliferation. Based on these considerations, its expression principally in pre-B1, -B2, and CBs might be consistent with its function as a metabolic regulator. A further gene co-expression module identified by this study consisted of genes that are expressed mostly in CCs and CBs or absent/downregulated in the GC, including several lincRNAs genes. Among them, they called attention to *RP11-132N15.3,* a lincRNA primarily expressed in CBs and to some extent in CCs. *RP11-132N15.3* gene is located approximately 240 kb upstream of *BCL6* on chromosome 3. The transcriptional repressor BCL-6 is known to control a great number of target genes involved in multiple signaling pathways that modulate and promote the GC reaction [28]. Overall, these genes act together to increase the threshold of response to DNA damage, which enables genetic modifications of Ig genes, impairs premature activation of B-cells, and blocks their final differentiation to allow the production of high affinity antibodies.

Among the few lncRNAs reported as differentially expressed during B-cell differentiation, we should mention the B-cell integration cluster (*BIC*), although a clear proof of *BIC* acting as a “long” RNA has not been found yet [29, 30]. *BIC* consists of three exons spanning a 13 kb region at chromosome 21q21 and shows high expression level in antigen receptor stimulated B- and T-cells as well as in macrophages and dendritic cells upon Toll-like receptor stimulation. Since the processed products of this lncRNA, miR-155-5p and miR-155-3p, have a central role in several biological processes, such as hematopoiesis, inflammation and immune responses [31], the studies related to *BIC* have been mainly focused on these miRNAs. Based on large-scale cloning studies [32], *BIC* has now been called MIR155 host gene or *MIR155HG* (http://www.genenames.org/) whereas the *BIC* transcript is identified as pri-miR-155. Notably, high expression levels of *BIC* and miR-155 have been also found in many types of mature B-cell malignancies including diffuse large B-cell lymphoma (DLBCL) and chronic lymphocytic leukemia (CLL) [31].

## LYMPHOMAGENESIS

B-cell malignancies are a heterogeneous group of neoplasms derived from mature or immature B-cells, and their phenotypic and biological characteristics largely mirror the normal development and maturation stages of B-cells. Similarly to most tumors, the coding genomes of malignant B-cells have numerous genetic aberration comprising deletions, amplifications and non-synonymous mutations. In addition, lymphomagenesis is characterized by the frequent occurrence of chromosomal translocations and aberrant somatic hypemutation, which are both related to the immunoglobulin remodeling mechanism including VDJ recombination, somatic hypermutation and class-switch recombination. Indeed, VDJ somatic recombination is carried out by two enzymes encoded by recombinase activating genes (*RAG1* and *RAG2*) that break double stranded DNA, normally followed by the DNA repair process that resolves and fixes the breaks. However, such breaks can contribute to chromosomal translocation in lymphoma. In addition, the function of the physiological somatic hypermutation mechanism in malignant B-cell may be aberrantly extended to non-immunoglobulin genes that are not targeted in healthy GC B-cells. In line with this, the genome of different subtypes of GC-derived lymphomas presents different recurrent primary chromosomal translocations and aberrant somatic hypermutations contributing to their pathogenesis [28] (Figure 2).

## LNCRNAS IN B-CELL MALIGNANCIES

The raising importance of lncRNAs in all types of cancer, suggests that they might be protagonist in the pathogenesis of B-cell malignancies. Although thus far only few lncRNAs have been identified and extensively characterized, the list is expected to grow exponentially in the near future (Table 2 and Figure 2).

**Table 2:** Most frequently deregulated lncRNAs in malignant B-cells

lncRNA(s)/alias	Disease type	Genome location	Functiona	Molecular mechanism	References
**BALR-1/c14orf132**	B-ALL	14q32.2	U	Not described	[35]
**BALR-2**	B-ALL	7q21.2	U	Inhibition of genes downstream of the glucocorticoid receptor (*FOS*, *JUN*, *BIM*)	[35]
**BALR-6**	B-ALL	3p24.3	U	Possible regulation of the transcriptome downstream of SP1	[35, 36]
**LINC00958/LOC1506305**	B-ALL	11q15.2	U	Not described	[35]
**DLEU2**	CLL, MM	13q14.3	TS	NF-kB activation. Host of miR-15a/16-1 cluster targeting *BCL2*	[42–48, 111]
**NEAT1**	CLL	11q13.1	TS	Induced by p53	[49, 52, 53]
**lincRNA-p21/TP53COR1**	CLL, DLBCL	17p13.1 (mouse) 6p21.2 (human)	TS	Induced by p53. Functionally linked to Cyclin D1, CDK4 and p21 in human DLBCL tissues	[49–51, 88]
**MIAT**	CLL	22q12.1	O	Constitution of a regulatory loop with OCT4	[54–57]
**ZNF667-AS1/lnc-AC004696.1-1**	CLL	19q13.43	U	Not described	[60, 61]
**BM742401**	CLL	18q11.2	TS	Not described	[58, 59]
**BIC**	CLL	21q21	OnP	Host of miR-155-5p and miR-155-3p	[29–31, 132, 133]
**lnc-IRF2-3**	CLL	4q35	U	Not described	[61]
**lnc-KIAA1755-4**	CLL	20q11.23	U	Not described	[61]
**GAS5**	MCL, DLBCL, MM	1q25.1	TS	Required for the inhibitory effects of mTOR antagonists. Regulated by mTOR pathway	[65–73, 111, 134]
**MALAT1**	MCL, MM	11q13	O	SP1 recruitment to the promoter of *LTBP3* gene regulating the bioavailability of TGF-β.	[74–76, 110–114]
**RP11-625 L16.3**	FL	12	U	Not described	[80]
**LOC283177**	DLBCL	11q25	U	Not described	[85]
**lnc-RP11-211G3.3.1-1**	DLBCL	3q27.3	U	Not described	[84]
**LUNAR1**	DLBCL	15q26.3	O	Regulated by NOTCH1. Enhancement of IGF1R mRNA expression	[86, 87]
**PEG10**	DLBCL	7q21.3	O	Activated by c-MYC	[89–91]
**HULC**	DLBCL	6p24.3	O	Not described	[92–95]
**HOTAIR**	DLBCL	12q13.13	O	Regulation of the PI3K/AKT/NF-κB pathway	[96]
**MINCR**	BL	8q24.3	U	Induced by MYC	[103]
**MEG3**	MM	14q32.2	TS	Interaction with p53. Regulation of *P53* gene expression. Enhancement of the expression of *BMP4* gene in MM-MSCs.	[115–117]
**TUG1**	MM	22q12.2	O	PRC2 binding to repress cell-cycle regulation genes. Induced by p53	[112, 118]
**lnc-SENP5-4/NCBP2 -AS2**	MM	3q29	U	Not described	[111]
**lnc-CPSF2-2**	MM	14q32	U	Not described	[111]
**lnc-LRRC47-1/TP73-AS1**	MM	1p36	U	Not described	[111]
**lnc-ANGPTL1-3**	MM	1q25	U	Not described	[111]
**lnc-WHSC2-2**	MM	4p16.3	U	Not described	[111]

*Abbreviations*: B-ALL, B acute lymphoblastic leukemia; CLL, chronic lymphocytic leukemia; BL, Burkitt lymphoma; DLBCL, diffuse large B-cell lymphoma; FL, follicular lymphoma; MCL, mantle cell lymphoma; MM Multiple Myeloma.

^a^ U = Uncharacterized; O = Oncogene; TS = Tumor Suppressor; OnP = Oncomir Progenitor

### B-acute lymphoblastic leukemia (B-ALL)

B-acute lymphoblastic leukemia (B-ALL) is the most frequent infancy tumor and one of the main reasons of death associated with cancer in children and young adults. In B-ALL, mutations and translocations occurring in precursor B-cells result in dysregulated gene expression [33, 34]. To date, the comprehension of the molecular pathogenesis of B-ALL has greatly improved; however, the precise function of lncRNAs in the disease is still largely to be defined.

Fernando et al. [35] undertook a microarray study to define patterns of lncRNAs expression in three different subsets of B-ALL (t(12; 21), *TEL-AML1*; t(1;19) *E2A-PBX1*; and t(4;11) *MLL-AF4*) finding that lncRNAs expression segregated with cytogenetic subtypes. Among the four most differentially regulated lncRNAs (*BALR-1*, *BALR-2*, *BALR-6*, and *LINC00958*) high expression of *BALR-2* (B-ALL associated long RNA-2), encoded by a locus on chromosome 7q21.2, correlated with a worse overall survival (OS) and a reduced response to prednisone treatment in a large cohort of B-ALL cases. Knockdown of *BALR-2* resulted in growth inhibition and increased apoptosis of B-ALL cell lines both at baseline and after glucocorticoid treatment. On the contrary, constitutive expression of *BALR-2* resulted in enhanced cell proliferation and resistance to prednisone administration. To note, gene expression analyses of cells with knockdown of *BALR-2* revealed activation of several genes, such as *FOS*, *JUN* and *BIM*, involved in the glucocorticoid receptor signaling pathway in both human and murine B-ALL cell lines. These results suggest that *BALR-2* may act in promoting B-ALL cells survival *via* inhibition of genes downstream of the glucocorticoid receptor.

In another study [36], the same Authors focused on *BALR-6* (B-ALL associated long RNA-6), expressed at the highest levels in *MLL*-translocated cases. [35]. The genomic location of *BALR-6* in humans is at 3p24.3. *BALR-6* showed some similarities with *BALR-2* as silencing of *BALR-6* in human B-ALL cell lines led to decreased cell proliferation and increased apoptosis, whereas overexpression of *BALR-6* isoforms in both human and murine cell lines induced increased growth and a partial resistance to prednisolone treatment. Furthermore, *BALR-6* overexpression *in vivo* in murine BM transplantation elicited a significant increase in hematopoietic progenitors, indicating that its deregulation may induce developmental alterations. Gene expression analyses of cells with knockdown of *BALR-6* revealed enrichment for leukemia-associated genes and biological programs controlled by the transcription factor SP1. Luciferase reporter assays measuring the transcriptional activity of the promoter of two SP1 targets (*CREB1* and *p21*) in HEK 293T cells demonstrated a great increase for both the promoter following SP1 and *BALR-6* co-overexpression.

### Chronic lymphocytic leukemia (CLL)

CLL is the most frequent form of adult leukemia in Western countries [37, 38]. The characteristic mark of CLL is the clonal expansion of mature B-lymphocytes with a well-defined phenotype (CD19+, CD5+, CD23+, CD22 low, and low density of surface Igs), which can infiltrate multiple organs including lymph nodes, the BM, spleen, and liver. CLL has a highly heterogeneous clinical evolution, ranging from an indolent behavior in most patients not requiring treatment at diagnosis and possibly surviving for decades, to a rather rapid aggressive disease (nearly 30%) that requires treatment. These clinical differences have been associated with a number of biological markers, such as chromosomal aberrations (13q, 11q and 17p deletions and trisomy 12), mutational status of the V region of the heavy-chain locus of the Ig genes (*IGHV*), *TP53* disruptions, CD38 and ZAP-70 expression, largely investigated with prognostic purposes [39–41]. However, these parameters largely fail to elucidate the heterogeneity in the evolution of the patients. Sequencing of the full genome and exome has clarified the mutational scenery of CLL, even though the molecular mechanisms at the base of the origin and evolution of the disease remain largely unknown.

Concerning lncRNAs with a potential pathogenetic role in CLL, great attention has been devoted to the deleted in leukemia 1 (*DLEU1*) and 2 (*DLEU2*) lncRNAs, which are located in the critical region at chromosome 13q14.3 found to be deleted in more than 50% of CLL patients [42, 43]. *DLEU2* hosts the miR-15a/16-1 cluster, known to have a central role in the pathogenesis of CLL, in part by increasing the expression of the oncogene *BCL2* [44, 45]. Mir-15a and mir-16-1 precursors are within intron 4 of *DLEU2*, on the same chromosome strand. Mice deleted for the entire minimal deleted region within 13q14, comprising the *Dleu2* lncRNA, were able to develop clonal B-cell proliferations recapitulating the spectrum of CLL in humans, showing a stronger aggressive phenotype than miR-15a/16-1-deleted mice [46]. A remarkable inhibitory effect of *DLEU2* was observed on cellular growth and colony-forming ability of cancer cell lines in a miR-15a/16-1-dependent manner [47]. In addition, *DLEU2* expression negatively regulated the Cyclin D1 and Cyclin E1 protein levels in a way reliant on the expression of miR-15a/16-1. Besides the deletion at 13q14.3, Garding et al. [48] suggested that the tumor suppressor activity at this region is multifactorial and probably involves further genetic elements than miR-15a/16-1, providing insights into the epigenetic regulation of all the genes in that region. In particular, the Authors found *DLEU1* and the *DLEU2/Alt1* isoform significantly and frequently demethylated in CLL. These two lncRNAs upregulation is connected with the *in cis* transcriptional downregulation of *DLEU2* and miR-15a/16-1, and of a cluster of adjacent protein-coding tumor suppressor genes regulating NF-kB activity. Taken together, these findings have suggested that loss of *DLEU2* might contribute to CLL through the absence or functional loss of miR15a/16-1, although additional roles, as described above [48], for *DLEU2* in CLL pathogenesis might be postulated.

The participation of lncRNAs in the p53 pathway in CLL and lymphoma was firstly revealed by Blume et al. [49]. After triggering the p53-dependent DNA damage response or non-genotoxic activation of p53 they identified two lncRNAs, nuclear enriched abundant transcript 1 (*NEAT1*) and *lincRNA-p21*, as p53 targets in primary CLL and in a series of Burkitt's lymphoma cell lines. A strict p53 dependent regulation was proven for both the lncRNAs by deletion and knockdown of p53 in lymphoma cell lines and by showing p53 binding to the *NEAT1* promoter. Furthermore, exposure of primary CLL cells to nutlin-3, a MDM2 inhibitor, induced both *NEAT1* and *lincRNA-p21* transcription in a p53-dependent manner. *LincRNA-p21* was primarily identified in the mouse, located on the chromosome 17, ∼15Kb upstream and on the opposite strand to the gene encoding the cell cycle regulator CDKN1A (also known as p21) [50]. Its human ortholog (also known as *TP53COR1*) maps to chromosome 6p21.2, antisense and in close proximity of the *CDKN1A* gene [51]. *LincRNA-p21* was demonstrated to repress gene transcription upon p53 activation in mouse [50] and to intervene in p53-dependent transcriptional suppression in human HeLa cells and mouse embryonic fibroblasts [51]. *NEAT1*, encoded on chromosome 11q13.1, is kept in the nucleus where it constitutes the core structural element of the paraspeckle sub-organelles, nuclear domains implicated in mRNA nuclear retention [52]. Notably, it has been shown that *NEAT1* regulates the nuclear keeping of mRNAs encompassing inverted repeats (principally Alu sequences), that can shape double stranded RNA regions liable to adenosine-to-inosine editing, leading to translational repression [53]. These findings suggest that p53 activation may affect the expression of numerous genes at post-transcriptional level through *NEAT1*.

Recently, Sattari et al. [54] investigated the lncRNA myocardial infarction associated transcript (*MIAT*) in leukemia/lymphoma cell lines encompassing almost completely the hematopoietic cell lineages showing upregulation in lymphoid lineage with mature B-cell phenotype, including CLL and NHL. Furthermore, the analysis of *MIAT* expression level in primary CLL samples demonstrated higher incidence of *MIAT* upregulation in aggressive types of CLL defined by chromosome abnormalities (17p del > 11q23 del = Trisomy 12, compared to indolent form carrying 13q-deletion) and worst clinical outcome. *MIAT* was originally identified within a susceptible locus for myocardial infarction on chromosome 22q12.1 [55]. *MIAT* accumulates within the nucleus as RNA component of specific nuclear bodies where it may affect RNA splicing by binding to splicing factors and exert a regulatory effect on gene expression [56]. The mouse homologue of *MIAT*, *Gomafu*, has been reported to bind to *Oct4* gene, enhancing the expression of Oct4 transcription factor in embryonic stem cells. On the other hand, Oct4 binds to and positively regulates *Gomafu* transcription, thus constituting a regulatory feedback loop in the modulation of mouse embryonic stem cells pluripotency [57]. Similarly, *MIAT* was shown to constitute a regulatory loop with OCT4 in tumoral mature B-cell and both *MIAT* and *OCT4* were demonstrated to be essential for cell survival [54]. Overall, these findings suggest an involvement of MIAT in supporting proliferation of the malignant mature B-cells.

A lncRNA gene, *BM742401*, whose lower expression in patients affected by gastric cancer was correlated with poor survival [58], was recently reported as frequently methylated in CLL [59]. More specifically, the promoter of *BM742401* was unmethylated in normal CD19-sorted peripheral B-cells, but methylated in four out of five CLL cell lines and in about 50% of primary CLL samples at diagnosis. Methylation of *BM742401* in CLL cell lines was inversely correlated with expression; treatment with a hypomethylating agent led to promoter demethylation and re-expression of *BM742401* transcript. Interestingly, in CLL samples *BM742401* methylation was significantly associated with higher lymphocyte counts and correlated with the advanced Rai stage (≥ stage 2), which represent poorer prognostic factors in CLL. Stable overexpression of *BM742401* in a cell line with methylated *BM742401,* resulted in inhibition of cellular proliferation and enhanced apoptosis, suggesting a tumor suppressive role of *BM742401* in CLL. However, the exact mechanism of tumor suppressive function of this lncRNA, localized to chromosome 18q11.2, is not fully understood and warrants further studies.

Besides investigations on selected candidates, only a few studies on global lncRNA expression have been performed in CLL. Ferreira et al. [60] performed deep RNA-sequencing in a cohort of 98 patients identifying 1089 genes differentially expressed in normal (naïve and memory B-cells) and tumor samples. In addition to protein-coding genes, the differentially expressed transcriptional elements included 127 lncRNAs, of which 47 were lincRNAs, and 61 pseudogenes [60]. More recently, Ronchetti et al. [61] looked for the lncRNA expression profiles in a wide prospective cohort of 217 Binet A patients and various subtypes of healthy B-cells, derived from tonsils or peripheral blood lymphocytes. All samples were profiled on Affymetrix Gene 1.0 ST array. LncRNA expression was revealed applying a custom pipeline capable to re-annotate the probes comprised in array based on the LNCipedia-v3.1 database. This strategy enabled the investigation of the expression levels of nearly two thousand well-annotated and specific human lncRNAs. They recognized a signature composed of 24 lncRNAs specifically dysregulated in CLL in respect to the healthy B-cell counterpart, such as naïve and memory B-cells. Furthermore, characteristic lncRNA signatures were identified in subgroups of patients prognostically stratified relying on cellular, molecular and cytogenetic markers, such as unmutated (UM) *IGHV* status, CD38 expression, 11q and 17p deletions, and *NOTCH1* mutations. Among lncRNAs frequently associated with the presence of unfavorable prognostic markers, it deserves to be mentioned *lnc-AC004696.1-1*, also noted as *ZNF667-AS1*, which is localized at 19q13, antisense and head to head to *ZNF667* gene. This lncRNA exhibited the highest expression in UM-CLLs, as previously shown by Ferreira et al. [60], and was negatively related to a short progression-free survival (PFS). The positive correlation of *ZNF667-AS1* with its antisense overlapping gene *ZNF667* prompts a possible role in *cis* regulation. Interestingly, ZNF667 was demonstrated to be a transcriptional regulator of the antiapoptotic gene *Bax* in rat [62]. Finally, the study by Ronchetti et al. proposed an independent risk model, based on the expression of two lncRNAs, *lnc-IRF2-3* and *lnc-KIAA1755-4*, able to differentiate three prognostic groups in the set of early-stage CLL samples investigated. Specifically, the model identified a “high-risk” group in which both lncRNAs were simultaneously expressed, whereas a “low-risk” group with the better PFS included the patients with a concomitant low expression of both lncRNAs. The divergent expression level of the two lncRNAs characterized the “intermediate-risk” group. To note, the model was shown to be independent from other known predictive factors in CLL and from a recently defined progression-risk score [63].

### Mantle cell lymphoma (MCL)

Mantle cell lymphoma (MCL) is a relatively uncommon, B-cell NHL predominantly affecting men older than 60 years. Although an aggressive disorder, it can have a more indolent course in some patients. These lymphomas are most likely to arise from early B-cells in the outer edge (mantle zone) of the lymph node follicle and most harbor the t(11;14)(q13;q32) translocation leading to the overexpression of Cyclin D1 [64]. MCL cells are typically CD5+ and CD23-, with high density of surface IgM/D. To date, one of the few noteworthy studies investigating the role of lncRNAs in MCL has based its assumptions on the observation that inhibition of the mammalian target of rapamycin (mTOR) is a strategic and hopeful way to treat MCL, which frequently shows activation of and dependence on the mTOR pathway for cell proliferation and survival [65]. Specifically, Mourtada-Maarabouni and Williams [66] used RNA interference in MCL cell lines to downregulate the lncRNA Growth Arrest Specific (*GAS5*), a cell-cycle arrest and apoptosis-related lncRNA with tumor suppressor activity [67–71], based on the fact that it is a known effector of the mTOR pathway in normal and transformed T lymphocytes [72, 73]. In line with this, also in MCL cells *GAS5* downregulation markedly diminished the effects of each mTOR antagonists on cell viability, DNA synthesis, and colony-forming power. These findings indicate that regulating *GAS5* may provide a new target for improvements in chemotherapy exploiting the dependence of MCL cells on the mTOR pathway.

Recently, Wang et al. [74] investigated the role in MCL of metastasis-associated lung adenocarcinoma transcript 1 (*MALAT1*), a putative oncogenic lncRNA of more than 8000 nucleotides transcribed from chromosome 11q13 and overexpressed in several solid tumors [75, 76]. They detected *MALAT1* overexpression in human MCL tissues and cell lines in confront with normal B-cells, showing a correlation with high International Prognostic Index (IPI) and lower OS of MCL patients. Knockdown of *MALAT1* hampered cell proliferation and enhanced apoptosis rates of MCL cell lines. Furthermore, Wang et al. demonstrated a raised expression of *CDKN1A/p21* and *CDKN1B/p27* after *MALAT1* silencing, whereas the levels of EZH2 and H3K27me3 protein expression were both reduced. The Authors suggested a *MALAT1* signaling in MCL in which *MALAT1* recruits the PRC2 complex by binding to EZH2 finally targeting *CDKN1A/p21* and *CDKN1B/p27* genes. p21 and p27 expression is then epigenetically repressed by H3K27me3. The consequent activation of cyclin-dependent kinase 1 and 2, due to p21 and p27 downregulation, promotes the phosphorylation of EZH2 further increasing its binding to *MALAT1*, which in turn enhances EZH2-mediated H3K27me3, and *p21* and *p27* genes repression. The result is an uncontrolled cell proliferation, providing *MALAT1* of some oncogenic properties.

### Follicular lymphoma (FL)

Follicular lymphoma (FL) is the second kind of NHL by frequency, generally characterized by a heterogeneous clinical course. From a biological point of view, FL cells are malignant counterparts of normal GC B-cells. Based on the relative ratio of CCs and CBs, FL grades 1 to 3 are recognized with a further distinction of grade 3A and 3B [77]. FL grades 1 to 3A have common histologic and molecular characteristics and an indolent clinical course, whilst FL grade 3B histologically looks like DLBCL, shows different molecular features and is clinically more aggressive. The genetic trait of FL is the translocation t(14;18)(q32;q21), resulting in the constitutive overexpression of the antiapoptotic BCL2 protein, which confers a survival advantage to FL cells. Although dysregulated BCL-2 expression appears to be fundamental in the pathogenesis of FL, it does not explain the entire pathogenesis of the disease. Even other chromosomal aberrations or gene mutations found in FLs, such as the translocations involving the *BCL6* gene locus [78] or the *EZH2* mutations [79], are not sufficient for explaining the mechanisms involved in lymphomagenesis.

In a recent study of lncRNA expression profiling by microarray, Pan et al. compared three FL3A with three reactive lymphatic nodes (RLN) [80]. They found statistically significant the upregulation of 152 lncRNAs and the downregulation of 37 lncRNAs comparing the FL3A and RLN groups. Four of the dysregulated lncRNAs were then validated by qRT-PCR in an independent group of 5 FL3A patients and 5 RLN. The most interesting finding was that *RP11-625 L16.3* (an antisense lncRNA located on chromosome 12) was significantly upregulated in FL3A patients, even if its biological functions are not clear yet.

### Diffuse large B-cell lymphoma (DLBCL)

DLBCL is the most frequent form of NHL in adults and represents a biologically heterogeneous and clinically aggressive group of disease entities classified based on morphology, immunophenotype, genetic aberrations, and clinical ongoing [77]. The majority of DLBCLs arises from antigen-exposed B-cells during the GC reaction, a process that optimizes the affinity of antibodies for antigens [81]. According to the cell of origin transcriptional classification, which relates subsets of DLBCL to specific stages of normal B-cell development, this type of lymphoma can be assigned to either a germinal center-B (GCB) or activated-B-cell (ABC) subtype [82]. The GCB subtype is preferentially diagnosed in young patients with a clinical outcome more favorable than the ABC subtype. Despite significant progress in diagnosis and treatment, a wide number of DLBCL patients still experiences a poor prognosis outcome. Thus, searching for novel molecular biomarkers associated with DLBCL pathogenesis, including lncRNAs, has been the aim of an increasing number of studies in recent years. As a result, a crucial involvement in the molecular etiology of DLBCL, as well as a potential relevance as markers of poor prognosis and therapeutic targets of the disease, have been suggested for different lncRNAs

Chromosome 1q25.1 has been associated with DLBCL because of recurrent breakpoints or duplication events [69, 83]. Notably, encoded at 1q25.1 locus is *GAS5* (see above), which was identified as a new non-Ig partner gene deregulating *BCL6* expression in a DLBCL case presenting the t(1;3)(q25;q27) [69]. In this patient, the *GAS5*-*BCL6* fusion transcripts were expressed in a preferential manner from the derivative chromosome 3, instead of *BCL6* from the normal allele.

Lu et al. [84] found that *lnc-RP11-211G3.3.1-1* gene in LNCipedia lncRNA database [1] is located in a region where the majority of the *BCL6* gene translocations breakpoints occurs. This lncRNA is transcribed antisense to *BCL-6* locus and, remarkably, its boundaries match those of the *BCL6* translocation zone quite precisely. Moreover, this lncRNA is a natural transcript in human DLBCL: expression of *lnc-RP11-211G3.3.1-1* was detected in the GBC subtype of DLBCL and at a lower level in the ABC subtype (Griffith M, Marra MA. http://www.alexaplatform.org/alexa_seq/DLBCL_ABC_GCB/genes/chr3_6/ENSG00000223401.htm. Accessed August 24, 2015).

Conde et al. [85] carried out a copy number variation analysis of 681 NHL patients and 749 controls, to correlate frequent structural alterations and lymphoma susceptibility. The Authors reported that the partial duplication of the 3′-region of the *LOC283177* lncRNA located at 11q25 could be associated with DLBCL risk. While the biological significance of *LOC283177* in DLBCL remains to be investigated, they suggested that it could be a possible susceptibility locus for the disease. Remarkably, in the younger DLBCL group *LOC283177* was associated with risk at a slightly higher degree than when considering the entire set of DLBCL patients. The duplication seems then to be more distinctive of the GCB than of the ABC DLBCL subtype; however, these findings need to be validated independently.

Peng et al. [86] investigated the role of lncRNA *LUNAR1* (LeUkemia-induced Non-coding Activator RNA-1) in DLBCL. This lncRNA is located at 15q26.3 and was firstly identified in human primary T-cell acute lymphoblastic leukemia (T-ALL) where it is upregulated in *NOTCH1* mutated cases. Furthermore, it is essential for efficient T-ALL growth *in vitro* and *in vivo* due to its ability to enhance the expression of its neighboring coding gene, *IGF1R*, thus sustaining the IGF1 signaling [87]. The levels of *LUNAR1* expression in DLBCL patient tissues and in six DLBCL cell lines were higher than in normal specimens. A significant correlation of the greater expression of *LUNAR1* with stage, rituximab treatment, and IPI was observed. Furthermore, *LUNAR1* expression functioned as an independent predictor for OS and PFS. *LUNAR1* silencing significantly inhibited cell proliferation of OCI-LY-7 DLBCL cell line, arresting the cells at the G0-G1 phase, and E2F1, Cyclin D1 and p21 were identified as functional targets of *LUNAR1* in DLBCL.

The same group [88] identified *lincRNA-p21* (see above) as a possible new prognostic biomarker/indicator of poor prognosis in DLBCL. *LincRNA-p21* expression evaluated by PCR in pathological tissues and cell lines was markedly lower than in healthy specimens, and the level of expression was significantly linked to staging, B symptoms, performance status, IPI score and serum LDH. Cases with low *lincRNA-p21* expression showed poorer OS and PFS rates. When ectopically expressed in OCI-LY7 cell line, *lincRNA-p21* repressed cellular growth and blocked cell cycle progression at the G0-G1 phase. Furthermore, high level of *lincRNA-p21* downregulated Cyclin D1 and CDK4 expression and upregulated p21, as assessed by Western blot analysis in agreement with the evidence in human DLBCL tissues. Overall, these findings indicate that *lincRNAp21* may act as a tumor suppressor possibly contributing to DLBCL development in case of lacking/reduced expression.

Peng et al. [89] investigated also the expression levels of lncRNA *PEG10* (paternally expressed 10), encoded from an imprinted gene located on chromosome 7q21 [90], in a series of DLBCL patients and cell lines. *PEG10* was found upregulated in DLBCL tumor tissues and cell lines matched with normal tissues and significantly correlated with B symptoms, IPI score, CHOP-like treatment and rituximab. The Authors proved that *PEG10* was an important predictive factor for diagnosis and prognosis of the disease on the base of the follow-up of a large group of patients. *PEG10* knockdown in RCK-8 cell line inhibited cell proliferation and promoted apoptosis. To note, *PEG10* was described as a direct transcriptional target of c-MYC in pancreatic and hepatocellular carcinoma cells [91]. Finally, Peng et al. [92] analyzed the expression levels of lncRNA *HULC* (highly up-regulated in liver cancer) in a cohort of DLBCL patients and cell lines and in 60 controls with reactive lymph nodes. This lncRNA, located at 6p24.3 has been found strongly overexpressed in human hepatocellular carcinoma [93, 94] and pancreatic cancer [95]. The expression levels of *HULC* in DLBCL patient tissues and in six DLBCL cell lines were significantly higher than in control specimens. Moreover, the greater expression of *HULC* was correlated with clinical stages, B symptoms, CHOP-like treatment, rituximab and IPI. To note, Peng et al. verified the importance of *HULC* as predictive factor for DLBCL diagnosis and prognosis in a sizable set of patients. *HULC* knockdown in SU-DHL-4 cell line significantly inhibited cell growth and promoted apoptosis by repressing Cyclin D1 and BCL2 proteins expression implicating a potential role for *HULC* as a therapeutic target in DLBCL.

The lincRNA *HOTAIR* (Hox transcript antisense intergenic RNA), encoded on chromosome 12q13.13, was found upregulated in DLBCL tumor samples, compared with normal tissues [96]. The upregulation of *HOTAIR* correlated with certain critical clinico-pathological characteristics, including clinical stage, B symptoms, IPI scores and tumor mass and predicted a poor prognosis in these patients. *HOTAIR* knockdown in RCK-8 cell line expressing high levels of this lncRNA, significantly reduced cell proliferation, arrested the cell cycle in the G2/M phase and stimulated cell apoptosis, probably through the PI3K/AKT/NF-kB pathway.

To date only a few studies concerning global lncRNA expression have been reported in DLBCL. Sun et al. analyzed lncRNAs expression profiles and clinical characteristics in a large cohort of 1043 DLBCL patients identifying prognostic lncRNAs that were significantly associated with OS [97]. Based on microarray expression data, they constructed a six-lncRNA signature able to classify DLBCL patients into high- and low-risk subgroups with significantly diverse survival outcome, independently of all other clinical factors conventionally considered for survival prediction of patients. Furthermore, the six-lncRNA signature was capable to predict effectively the survival outcome of DLBCL patients with similar IPI variables. Specifically, patients with low lncRNA expression-based risk score tended to express five lncRNAs (*MME-AS1*, *CSMD2-AS1*, *RP11-360F5.1*, *RP11-25K19.1* and *CTC-467M3.1*), whereas *SACS-AS1* was upregulated in patients with high risk score. Functional enrichment analysis of protein-coding genes co-expressed with prognostic lncRNAs suggested that the six-lncRNA signature might be implicated in regulatory functions of known cancer-related pathways and immune system-related biological processes closely linked with the mechanisms of lymphomagenesis and progression of DLBCL.

A transcript discovery strategy set up on RNA-sequencing of 116 primary tumors and normal B-cell specimens was used by Verma et al. to identify and characterize novel lncRNAs in DLBCL [98]. They recognized 2,632 new lncRNAs expressed in more than one patient, two-thirds of which were absent in normal B-cells. Many lncRNAs had more than one isoform, and more than one-third were differentially expressed in the ABC and GCB DLBCL subtypes. Correlations with protein-coding genes showed that > 80 % of lncRNAs were significantly co-expressed with at least one gene, suggesting possible co-regulatory mechanisms. Pathway analysis on the array of co-expressed coding genes for each lncRNA revealed that 43% of the lncRNAs showed enrichment for significant DLBCL-related functional pathways, such as CD40 pathways.

### Burkitt lymphoma (BL)

Burkitt lymphoma (BL) is an aggressive GC B-cell-derived NHL that affects mostly children and young adults, particularly males [99]. BL is subdivided into three different categories based on epidemiological observations: endemic (rare outside Africa), sporadic (occurring throughout the world), and immunodeficiency-associated [77]. Endemic BL is frequently associated with the Epstein-Barr virus (EBV) infection. Immunodeficiency-related BL is diagnosed predominantly in patients infected with HIV/AIDS, but can also occur in people who have inherited immune deficiencies or those who underwent organ transplant. The molecular hallmark of BL is translocation of the *MYC* oncogene, juxtaposing the *MYC* locus at 8q24 and one of three immunoglobulin loci. Eighty percent of BL patients have t(8;14) that juxtaposes the *MYC* gene with *IGH* enhancer elements on chromosome 14, resulting in the *MYC* constitutive transcriptional deregulation. In the remaining 20% of BL tumors, translocations t(2;8) or t(8;22) place the *MYC* gene adjacent to either kappa or lambda light chain loci and enhancer elements, respectively. Even though the constitutive expression of MYC is considered the chief responsible for BL development, deregulation of this transcription factor by juxtaposition with the immunoglobulin loci seems not to be sufficient to drive lymphomagenesis. Indeed, further genetic lesions cooperate with *MYC* to generate the disease, as revealed by high-throughput sequencing approaches [100–102]. Furthermore, since MYC protein is central in many tumors by regulating the expression of thousands of target genes, it could also modify the expression of lncRNAs participating in oncogenic transformation.

In order to identify MYC-regulated lncRNAs possibly involved in lymphomagenesis, Doose and colleagues examined RNA-sequencing data of patients from the major subtypes of mature B-cell lymphomas, namely BL, DLBCL, and FL, compared with data from healthy GC B-cells. The results were then crossed with those deriving from cell lines expressing inducible MYC to identify MYC-regulated lncRNAs [103]. They identified 13 lncRNAs differentially expressed in IG-MYC-positive BL relative to normal GC B-cells and concordantly regulated by MYC in the model cell lines. In particular, they focused on the lncRNA that had the highest positive correlation with MYC expression in MYC-positive lymphomas, which they titled MYC-induced long noncoding RNA (*MINCR*). The *MINCR* gene is intergenic to the *GLI4* and *ZNF696* coding genes at chromosome 8q24.3. *MINCR* knockdown was associated with reduced cellular proliferation in three different cell types. The investigation of gene expression changes induced by *MINCR* knockdown in the cell line where the effect was most pronounced (hT-RPE-MycER), showed a significant enrichment of genes involved in cell cycle initiation and progression among those downregulated. These genes showed a significant enrichment of MYC-binding sites in their promoters, suggesting that *MINCR* may act as a modulator of the MYC transcriptional program. This model was supported by the observation that the promoters of selected cell cycle genes decreased MYC binding following *MINCR* knockdown.

Another study investigating the P493-6 B-cell line carrying an inducible *MYC* allele and primary *MYC*-associated B-cell lymphoma samples, showed that lncRNAs are the principal element of the MYC-regulated transcriptional program [104]. In total, more than one thousand lncRNA loci were identified as regulated by MYC in the P493-6 model. Both MYC-induced mRNAs and lncRNAs but only MYC-repressed lncRNAs were significantly enriched for MYC binding sites. In addition, the Authors analyzed lncRNA differentially expressed in B-cell lymphoma with high and low MYC levels. Among them, a rate greater than 50% was even identified as MYC-regulated in P493-6 cells and was responsive to *MYC* knockdown in BL cell lines. All these data sustain the potential pathogenetic relevance of these lncRNAs.

### Multiple myeloma (MM)

Multiple myeloma (MM) is a fatal malignant proliferation of antibody-secreting BM PCs that accounts for 10% of all hematological malignancies. MM is characterized by a wide clinical spectrum ranging from a pre-malignant condition called monoclonal gammopathy of undetermined significance (MGUS), to extra-medullary myeloma/plasma cell leukemia (PCL) [105–107]. MM shows a severe genomic instability leading to both aberrant ploidy and structural rearrangements [105, 108]. Virtually half of MM tumors are hyperdiploid, the remaining ones are frequently associated with the constitutive activation of *CCND1* (11q13), *CCND3* (6p21), *MAF* (16q23), *MAFB* (20q11), or *FGFR3*/*MMSET* (4p16.3) genes, as a result of chromosomal translocations involving the *IGH* locus on chromosome 14q32. Despite the remarkable improvements in treatment and patient care [109], MM remains an incurable disease.

Data concerning lncRNAs involvement in MM are expanding. The first lncRNA found to be overexpressed in BM mononuclear cells of MM patients was *MALAT1*. Specifically, Cho et al. [110] found *MALAT1* expression significantly higher in MM patients at diagnosis compared to treated patients or healthy individuals. In addition, patients who experienced disease progression or relapse showed a significant increased expression of *MALAT1*. A recent study by Ronchetti et al. [111], investigating lncRNA expression profiles in a large cohort of patients representing all the major different forms of PC dyscrasias, confirmed and extended these finding. Interestingly, *MALAT1* upregulation was associated with molecular pathways related to cell cycle regulation, p53-mediated DNA damage response, and mRNA maturation processes. Differently from the evidence of *MALAT1* overexpression in MM cells [110, 111], Isin et al. reported that circulating levels of *MALAT1* transcripts were significantly lower in MM patients compared to healthy subjects [112]. Finally, it has been recently demonstrated that *MALAT1* is overexpressed in mesenchymal stem cells (MSCs) from MM patients and regulates the transcription of the nearby antisense protein-coding gene *LTBP3* (latent TGF-β-binding protein) [113], known to positively regulate the activity of TGF-β, which may contribute to the inhibition of terminal osteoblastogenesis in MM [114]. More specifically, *MALAT1* was shown to recruit the transcription factor SP1 on the *LTBP3* promoter contributing to the increase of *LTBP3* expression. Notably, knockdown of *MALAT1* significantly decreased *LTBP3* transcription [113].

Maternally expressed gene 3 (*MEG3*), encoded at 14q32.2, is a lncRNA with a tumor suppression function mediated by both p53-dependent and p53-independent mechanisms [115], whose expression is epigenetically regulated. Benetatos et al. [116] studied the expression of *MEG3* in the context of MM and found that about 60% of patients presented hypermethylation of the differentially methylated region (DMR) of the *MEG3* promoter. A correlation with the disease stage was observed as well as with the disease subtype; notably, 67.7% of the patients with IgG MM and 100% of the IgM MM had hypermethylated DMR, whereas none of the patients with IgA MM presented the specific epigenetic change. It was therefore suggested that promoter hypermethylation of the *MEG* lincRNA and the consequent decreased expression might be involved in MM tumorigenesis. Interestingly, MSCs from MM patients (MM-MSCs) showed *MEG3* expression levels lower than those from normal donors (ND-MSCs) during osteogenic differentiation [117]. Furthermore *MEG3* overexpression in MM-MSCs could promote their osteogenic differentiation, whereas *MEG3* knockdown compromised osteogenesis of ND-MSCs. Experimental data suggested that *MEG3* favors osteogenesis by targeting the transcription of *BMP4* (Bone morphogenetic protein 4) gene. *MEG3* and *BMP4* both map on chromosome 14q but in opposite transcriptional orientation. In the proposed model, *MEG3* may directly interact with the transcription factor SOX2 causing its detachment from the *BMP4* promoter and finally increasing the expression of *BMP4* [117].

Preliminary evidence has also suggested deregulated circulating levels of a few selected lncRNAs in MM patients compared to healthy donors (HD) [112]. A study by Isin et al. investigating the circulating levels of selected lncRNAs in plasma of patients with B-cell malignancies found that expression of the lncRNA taurine upregulated gene 1 (*TUG1*), transcribed from chromosome 22q12.2, was significantly different in plasma of patients with MM compared to healthy subjects [112]. Furthermore, higher levels of *TUG1* correlated with disease state in both MM and CLL. *TUG1* has been shown to be transcriptionally regulated by p53 in response to DNA damage and to be involved in repressing important cell cycle related genes by recruiting the Polycomb Repressive Complex 2 (PRC2) at chromatin level [118].

Besides studies on selected candidates, only few studies on lncRNA expression at genome-wide transcriptome level have been performed in MM. In particular, Zhou et al. [119] investigated a repertoire of 2330 lncRNA from a large cohort of 559 MM patients by re-annotating different publicly available microarray datasets and developed a four-lncRNA prognostic signature predicting survival in MM patients. Notably, two of these lncRNAs were located respectively to chromosome 1p (*RP1-43E13.2*) and 1q (*RP4-803J11.2*) which are found to be lost or gained in a significant fraction of MM and associated with an adverse prognosis. More recently, Ronchetti et al. [111], investigated global lncRNA expression profiles by new generation microarrays in a large cohort of 259 patients, representing all the major different entities of PC dyscrasias, including MGUS, asymptomatic smoldering MM (SMM), truly overt and symptomatic MM, and extra-medullary MM/PCL patients, in addition to nine HD. The Authors analyzed the expression of 1852 lncRNAs specified by unique probes on the array and found that 230 lncRNAs were able to well distinguish normal *versus* pathological samples as well as the different forms of PC dyscrasias. Furthermore, 31 lncRNAs, including *MALAT1* (see above), were found specifically deregulated in pathological samples compared to normal BM PCs. In addition, 21 lncRNAs displayed progressively deregulated expression in association with more aggressive form of the disease. Among these, *lnc-SENP5-4*, *lnc-CPSF2-2*, and *lnc-LRRC47-1* were found significantly differently expressed in a panel of 19 MMs at diagnosis compared to the corresponding relapse/PCL phases. Notably, a recent study investigating *lnc-LRRC47-1* in MM, confirmed its progressive downregulation from normal PCs to MGUS and symptomatic disease, which appeared independent from the methylation status of its promoter [120]. Furthermore, the 21 lncRNA list included *GAS5* and *lnc-ANGPTL1-3*, both located at 1q25, a finding in agreement with the GEP-model for high-risk MM characterized by the expression of many genes mapping to chromosome 1q [121]. Interestingly, *GAS5* was found significantly upregulated in MMs with 1q gain. Concerning other main genomic alterations in MM, Ronchetti et al. found a significant downregulation of *DLEU2* in patients carrying del13; in addition, *DLEU2* expression significantly correlated with that of miR-15a and miR-16-1. Considering MM patients carrying t(4;14), it is of relevance the upregulation of *lnc-WHSC2-2* that maps intronic and antisense to the translocation target gene *MMSET*. Notably, the consistent deregulation of *lnc-WHSC2-2* in translocated MM resembles that of snoRNA *ACA11* that maps in the adjacent 3′ intron of *MMSET* on the sense strand [122, 123]. It is conceivable that, as reported for *ACA11* [123], lnc-WHSC2-2 may be a critical target of the t(4;14) translocation in MM with a potential oncogenic role. Finally, the 1q-gain MM group showed significantly upregulated seven lncRNAs all located at 1q region, suggesting that gene dose effect may also be a mechanism behind lncRNAs deregulation. Notably, Ronchetti et al. extended the study on the role of lncRNA deregulation in MM, based on the emerging evidences of the cross-regulation between lncRNAs and miRNAs. In particular, they investigated in silico the miRNA/lncRNA relationship in MM and PCL tumors and in normal PC samples, by integrating expression data with target predictions. They identified lncRNA-miRNA pairs (*lnc-MCL1-2* and mir-17 gene family; *lnc-AGBL1-4* and mir-185-5p; *lnc-DLEU2* and miR-3175; *LINC00173* and miR-221) with potentially relevant interactions for MM biology, providing an interesting suggestion of the pathological impact of miRNA/lncRNA cross-talk in MM [124].

## LNCRNA-BASED THERAPEUTIC STRATEGIES

As discussed above several studies have revealed a high number of deregulated lncRNAs in B-cell malignancies, but only a few have been deeply characterized so far. Based on their correlation with prognosis, OS or drug-response, some lncRNAs prompt further investigation to be introduced in pre-clinical and clinical practice. Nowadays, lncRNA-based therapeutic approaches represent a challenging avenue of research largely heartened by the encouraging results obtained with miRNAs-based strategies. Indeed, miR-29b [125] mimics as well as miR-221/222 inhibitors are promising anti-MM therapeutic agents when administered *in vitro* and *in vivo* in human myeloma cell lines or in human MM xenografts mice [126, 127]. In line with this, the loss of function of distinct lncRNAs could be overcome by a replacement therapy that reintroduces such lncRNAs into cells as drugs, whereas when lncRNAs are upregulated in cancer, three main tools could be exploited to affect their expression: the use of short interfering RNAs (siRNAs), Antisense Oligonucleotides (ASOs), or locked nucleic acid (LNA) nucleosides. SiRNAs are double stranded DNA molecules that exactly bind a stretch of nucleotides, resulting in RNA target degradation. ASOs are single stranded molecules smaller than siRNAs and, therefore, able to enter more easily into the nucleus, where lncRNAs are often localized. ASO molecules were used *in vivo* and *in vitro* against *MALAT1* resulting in a reduction of lung cancer metastasis formation [128]. LNA nucleosides are nucleic acid analogues with the ribose ring “locked” by a methylene bridge. Thanks to this modification, LNA oligonucleotides demonstrate extraordinary hybridization affinity toward both RNA and DNA, with excellent mismatch discrimination. Finally, the genetic editing offers a valid alternative to lncRNAs knockout mediated by siRNA or ASO, by targeting the genomic DNA straight. The most recent genetic tool available for this purpose is the clustered regularly interspaced short palindromic repeats (CRISPR)/CRISPR-associated (Cas) system [129]. Notably, the CRISPR/Cas9 system and paired single guide RNAs (sgRNAs) is able to produce large fragment deletions.

In the next future, these approaches will be fundamental to discover the biological function of lncRNAs in cells. Data from these studies are arousing great interest among the scientific community and will be central for the future growth of this novel class of therapeutic agents [130, 131]. However, therapeutic applications of lncRNAs, as for RNA in general, must deal with considerable obstacles, including improvement of reliable delivery systems, dosage regimes and techniques to reduce off-target effects. Moreover, we should not overlook the problems related to targeting transcripts the size of lncRNAs due also to their extensive secondary structures. Finally, it is essential to develop more genetic model systems aimed at understanding the function of lncRNAs *in vivo.* Notwithstanding all these limits to be overcome, lncRNAs seem to be promising therapeutic targets.

## CONCLUSIONS

LncRNAs represent a very large and complex family of protein-non coding transcripts with an emerging pivotal role in many molecular and physiological processes associated with the biology of normal and cancer cells. As for miRNAs, also lncRNAs may have strong potential as novel diagnostic and prognostic biomarkers in cancer, mainly due to the evidence of their tissue-specific expression and their easy detection in body fluids, and may represent novel therapeutic agents or targets. However, the genomic annotation of lncRNAs is currently limited.

In this review, we summarized and discussed the current knowledge about normally expressed and dysregulated lncRNAs during B-cell differentiation and in B-cell malignancies that in many instances represent cells blocked at different stages of maturation. As it is the case of other types of cancer, the investigation of lncRNAs in B-cell malignancies is still in its infancy. Several studies have revealed a high number of lncRNAs that are deregulated in B-cell malignancies, but only a few have been extensively characterized, prompting the need for further investigations. Furthermore, the global expression profiling of lncRNAs in these malignancies is still limited; most of the available data have been generated by PCR or microarray based-studies and need to be validated in large series of patients and with more extensive use of innovative technical approaches such as RNA-sequencing. Based on these considerations, we believe that exploring the landscape and functions of lncRNAs in normal B-cells and in their malignant counterparts will represent a compelling and challenging task in the next future with an important biological and clinical impact in the field.
